# Origins and transformations of dissolved organic matter in large Arctic rivers

**DOI:** 10.1038/s41598-017-12729-1

**Published:** 2017-10-19

**Authors:** Karl Kaiser, Maria Canedo-Oropeza, Rachel McMahon, Rainer M. W. Amon

**Affiliations:** 1 Texas A&M University Galveston Campus, Department of Marine Sciences, Galveston, USA; 2 Texas A&M University, Department of Oceanography, College Station, USA; 3 Old Dominion University, Department of Ocean, Earth and Atmospheric Science, Norfolk, USA

## Abstract

Arctic river watersheds are important components of the global climate system and show an amplified response to climate change. Here, we characterize origins and transformations of dissolved organic matter (DOM) in five major Arctic rivers (Kolyma, Lena, Yenisei, Ob, Mackenzie) over 3 years with seasonal sampling periods using measurements of carbohydrates, amino acids, bacterial biomarkers (D-amino acids), and plant protein biomarkers (hydroxyproline). A strong seasonal cycle of bioavailable DOM export was observed that correlated with discharge, vegetation, river morphology and water residence time. The chemical composition of bioavailable DOM was different among rivers reflecting unique characteristics of Arctic river watersheds. Trends in specific bacterial biomarkers were synchronous to changes in bacterial community compositions demonstrating that bacterial communities responded to the seasonal shifts in organic matter quality and chemical composition. Extensive heterotrophic processing of plant and soil-derived DOM resulted in major inputs of bacterial detritus, and bacterial organic matter accounted for 21–42% of DOC in all watersheds. Dissolved organic nitrogen sources were dominated by bacterially-derived nitrogen and important contributions of soluble plant protein during the Spring freshet. Overall, our results demonstrated the importance of watershed characteristics and bacterial metabolism in regulating DOM composition, reactivity and carbon fluxes in Arctic river watersheds.

## Introduction

Arctic watersheds store approximately 50% of global soil organic carbon of which much is held in shallow continuous and discontinuous permafrost soils^1^. In addition, these watersheds hold a significant portion (10% or ~50 Pg C) of the global biomass in the form of vegetation^2^. Recent estimates for carbon fluxes in this region indicate that the large Arctic watersheds are currently net sinks for CO_2_ (200–400 TgC yr^−1^)^2^, net sources for CH_4_ (33–46 TgC yr^−1^)^2^, and deliver between 25 and 36 TgC yr^−1^ in the form of dissolved organic carbon (DOC) to the Arctic Ocean^3,4^. Although most studies agree that a warmer climate will intensify the carbon cycle in high latitude watersheds, estimates of future terrestrial carbon transport in these rivers are variable and uncertain. While some studies predict a several fold increase in high latitude river organic carbon transport, other studies indicate a reduction of organic carbon concentrations due to elevated respiration rates within the watersheds^5–7^.

Arctic river watersheds also integrate diverse vegetation and soil regimes with complex hydrological characteristics and various climate zones. The latitudinal vegetation gradient generally captures a transition of boreal forests (taiga) dominated by coniferous evergreen trees (gymnosperm) in the southern regions to tundra populated by lichens, mosses and angiosperm vegetation in the North. Above 50–60°N, discontinuous and continuous permafrost is widespread restricting soil drainage that leads to extensive development of peatlands especially in the Siberian river watersheds^2^.

Detailed information on DOM sources in Arctic rivers is limited. What is known is mostly based on measurements of lignin and hydroxy-benzene biomarkers, and optical parameters such as chromophoric dissolved organic matter (CDOM) absorbance and fluorescence^8–11^. Lignin and hydroxy-benzene biomarkers indicate that water-soluble components of fresh litter from angiosperm and gymnosperm plants are likely important sources of DOM in these rivers during the Spring freshet^10^. At low flow conditions in late summer, fall and winter, Arctic river DOM appears to carry a substantial fraction of DOM derived from mosses and peat bogs with an older radiocarbon signature indicative of deeper soil horizon drainage^9,12–14^.

Dissolved organic nitrogen (DON) constitutes the majority of total dissolved nitrogen (TDN) in Arctic rivers and potentially supports a substantial fraction of near-shore primary production^4,15^. DON in terrestrial environments is an operational definition for a structurally complex mixture derived from various sources^16^. Dominant N-containing compounds in DON are characterized by amide linkages found in amino acids and amino sugars, amine functional groups and aromatic structures of the pyrrolic and pyridinic type^17,18^. There have been few reports indicating a major bacterial contribution to riverine DON^19–21^. Dissolved amino acids are important components of DON in boreal streams^19^ and appear to be important constituents of bioavailable DOM in Arctic rivers^22^. Nitrogen-bearing biopolymers in plants are dominated by hydroxyproline-rich glycoproteins. These glycoproteins represent families of arabinogalactan proteins, extensins and solanaceous lectins and occur in the primary cell wall of all land plants and most green algae^23^.

The bioavailability of Arctic riverine DOM appears to be closely linked to its chemical composition and source. High bioavailability of Spring freshet DOM reflects the recent origin of DOM leached from fresh litter and surface soil horizons, abundant mineral nutrient, and little previous decomposition due to cold temperatures in soils^11,22,24^. DOM transported in the rivers after the freshet, when flow is dominated by groundwater, bears the signature of older, extensively decomposed organic matter from soils, peat- and wetlands and exhibits drastically lower bioreactivity^10,24^.

Mineralization of riverine DOM continues at the river-estuary interface, on the Arctic shelves, and the Arctic Ocean, and contributes to nutrient budgets, air-sea CO_2_ exchange, and acidification in coastal seas^25,26^. Microbial and photochemical mineralization removes about 50% of discharged riverine DOC per year on the shelves suggesting only a very small fraction survives in the ocean over centuries and millennia^26,27^. Yet the question about the importance of riverine DOC for the marine carbon cycle is still debated. A recent study by Zigah *et al*.^28^ reported a large allochthonous source of DOC in the ocean (~25% of DOC) possibly derived from terrestrial sources or from hydrothermal vent systems.

Bioassay incubations have shown amino acids and neutral sugars serve as biochemical indicators of the labile and semi-labile DOM reactive on timescales of hours to years^29–31^. These biomolecules are enriched in freshly-produced organic matter, and they are preferentially utilized during biodegradation. Here, we analyzed amino acids and neutral sugars including bacterial biomarkers (D-amino acids) and plant biomarkers (hydroxyproline) to examine seasonal sources and bioavailability of DOM in five major Arctic rivers (Ob, Yenisei, Lena, Kolyma, Mackenzie) over multiple years. Seasonal patterns observed among rivers reveal fundamental mechanisms that link chemical composition, bacterial community compositions, and watershed characteristics to transformations of DOM in Arctic watersheds and export to the coastal margins. Results of our study demonstrate bacterial metabolism plays an important role in the export of terrestrial carbon from Arctic watersheds.

## Results

**Figure 1:**
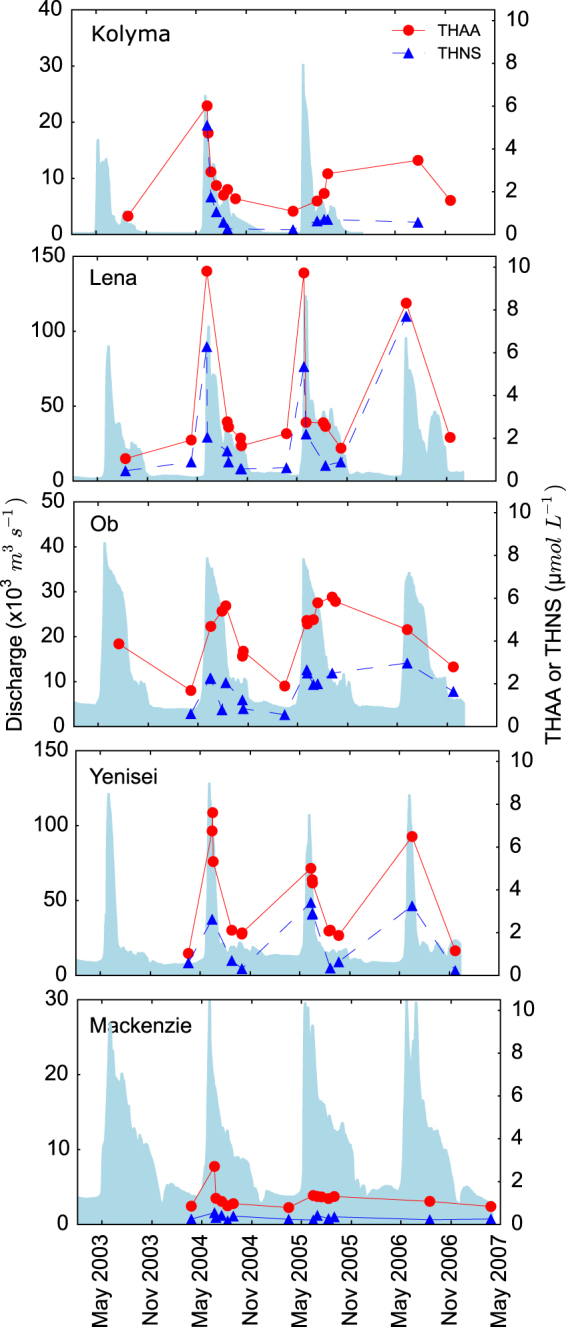
Seasonal discharge (m^3^ s^−1^) and concentrations (μmol L^−1^) of total hydrolysable amino acids (THAA) and total hydrolysable neutral sugars (THNS) in the Kolyma, Lena, Ob, Yenisei, and Mackenzie between 2003 and 2007. Light-blue shaded areas show discharge.

Concentrations of DOC, total hydrolysable amino acids (THAA), and total hydrolysable neutral sugars (THNS) exhibited pronounced changes connected to the seasonal rate of water flow in all Arctic rivers (Fig. 1, Table S1, Table S2). In general, peak discharge during each year was associated with highest concentrations followed by a substantial decline during summer, fall and winter seasons. In the Ob river, the highest concentrations of total hydrolysable amino acids (THAA) were observed at the end of the freshet whereas in other rivers highest concentrations occurred with the onset of the freshet. The Mackenzie showed much smaller concentrations changes of THAA and total hydrolysable neutral sugars (THNS) than the Siberian rivers.

Discharge-weighted average concentrations of THNS ranged from 359–4460 nmol L^−1^ during the Spring freshet, 300–1589 nmol L^−1^ during Summer/Fall, and 246–1168 nmol L^−1^ during Winter/early Spring. Discharge-weighted average concentrations of THAA ranged from 1585–6887 nmol L^−1^ during the Spring freshet, 1117–5281 nmol L^−1^ during Summer/Fall, and 824–2354 nmol L^−1^ during Winter/early Spring.

The five Arctic rivers exported a combined total of 17.23 TgDOC yr^−1^, 0.19 TgTHAA-C yr^−1^ and 0.22 TgTHNS-C yr^−1^ annually (Table 1). The Ob, Yenisei and Lena accounted for ~90% of DOC and organic carbon contained in THAA and THNS. Annually discharged DOC showed lowest THAA and THNS yields in the Mackenzie than any other river. The Kolyma discharged the most THNS enriched DOC (% OC) followed by the Ob and Yenisei (Table 1).

**Table 1:** Concentrations, yields, annual loads, and relative contributions of DOC and biochemicals.

	Kolyma	Lena	Ob	Yenisei	Mackenzie
DOC (μmol L^−1^)^1^	651 ± 71	1008 ± 66	842 ± 25	901 ± 50	396 ± 13
THNS (nmol L^−1^)^2,3^	2809 ± 1070	3726 ± 922	2066 ± 209	2711 ± 208	327 ± 40
THAA (nmol L^−1^)^2,4^	4079 ± 693	5375 ± 1194	4817 ± 181	5609 ± 463	1365 ± 163
D-AA (nmol L^−1^)^2,5^	187 ± 28	239 ± 57	227 ± 14	207 ± 14	82 ± 3
Hyp (nmol L^−1^)^2,6^	20 ± 9	36 ± 11	22 ± 7	13 ± 7	7 ± 2
THNS (%OC)^2^	2.2 ± 0.6	1.9 ± 0.4	1.4 ± 0.2	1.7 ± 0.2	0.5 ± 0.1
THAA (%OC)^2^	2.1 ± 0.2	1.8 ± 0.4	2.0 ± 0.1	2.2 ± 0.1	1.2 ± 0.1
DOC load (TgC yr^−1^)^7^	0.86	6.66	3.91	4.65	1.15
THAA (GgC yr^−1^)^7^	12	61	44	63	7
THNS (GgC yr^−1^)^7^	16	76	53	67	5
DOC total (%)^8^	5	39	23	27	7
THAA-C total (%)^8^	6	33	24	34	4
THNS-C total (%)^8^	7	35	25	31	3
Discharge yield_THAA_ (%OC)^9^	1.4	0.9	1.1	1.4	0.6
Discharge yield_THNS_ (%OC)^9^	1.8	1.1	1.4	1.4	0.5

^1^Concentrations of DOC were weighted to discharge.

^2^Concentrations and yields were weighted to discharge and DOC concentrations.

^3^THNS, total hydrolysable neutral sugars.

^4^THAA, total hydrolysable amino acids.

^5^D-AA, sum of D-Asx, D-Glx, D-Ser and D-Ala.

^6^Hyp, hydroxyproline.

^7^Annual loads of DOC, THAA-C and THNS-C was calculated using LOADEST with LoadRunner^61,62^.

^8^Relative contributions of each river.

^9^Carbon normalized discharge of biochemicals.

**Figure 2:**
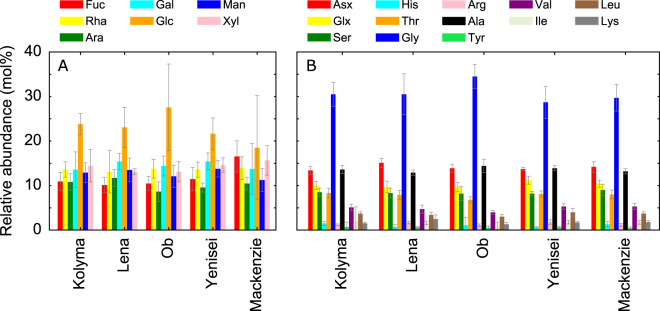
Average relative abundances (mol%) of (**A**) total hydrolysable neutral sugars (THNS) and (**B**) amino acids (THAA) for all flow regimes. See methods for abbreviations and Tables S1 and S2 for detailed data.

Mol percentages of THNS and THAA in all rivers were relatively uniform, although Arctic rivers integrated diverse vegetation and soil regimes with complex hydrological characteristics and various climate zones (Fig. 2, Figs S1, S2 Table S3). Compositions of THNS showed some variability during the different stages of the flow regime, although no clear trend was visible (Fig. S1). THAA were compositionally different during the Spring freshet compared to low flow conditions observed during Winter/early Spring showing strong enrichment in glycine during Winter in all rivers (Fig. S2). Compositions of amino acids during Summer/Fall reflected the transition between Spring freshet and base flow.

**Figure 3:**
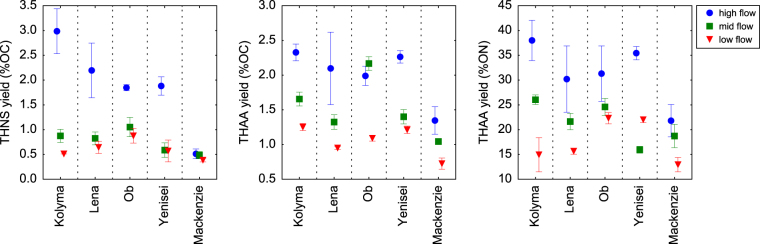
Carbon- and nitrogen-normalized yields of total hydrolysable neutral sugars (THNS) and amino acids (THAA) separated by different flow regimes.

Carbon-normalized yields of THNS and THAA were consistently highest during the Spring freshet in the Siberian rivers ranging from 1.8–3.0 and 1.3–2.3% DOC (Fig. 3) and indicating the presence of bioavailable DOM. In contrast, yields in the Mackenzie River did not change much during seasons. Both, THNS and THAA yields, were well correlated with total dissolved lignin phenol yields reported by Amon *et al*.^10^ (Fig. S3). Total dissolved lignin phenols (TDLP) included hydroxy, vanillyl, syringyl, and cinnamyl phenols.

Nitrogen-normalized yields of THAA followed trends observed for C-normalized yields in the Siberian rivers, showing 22–38% of DON was derived from amino acids during the Spring freshet, and 16–26% of DON during Winter/early Spring. Again, the Mackenzie showed only small changes between seasons (18 ± 5% DON).

**Figure 4:**
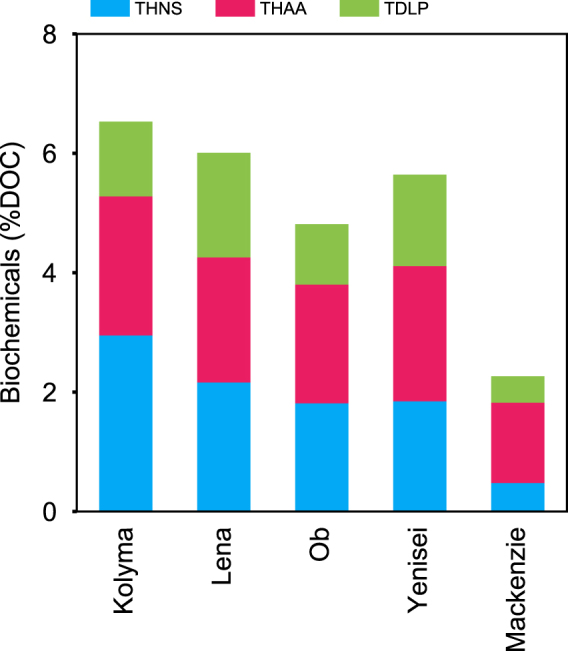
Carbon-normalized contributions of total hydrolysable amino acids (THAA), total hydrolysable neutral sugars (THNS), and total dissolved lignin phenols (TDLP) to dissolved organic carbon (DOC) during the Spring freshet. Data for TDLP was from Amon *et al*.^10^ and include hydroxy, vanillyl, syringyl, and cinnamyl phenols.

Together, THNS, THAA, and TDLP comprised 2–7% of freshet DOC in all rivers (Fig. 4). The percentage of DOC characterized by amino acids, neutral sugars and lignin phenols was ~3 times higher in the Siberian rivers compared to the Mackenzie. Freshet DOC in the Kolyma showed highest yields of carbohydrates among all rivers. Lignin phenol yields were lowest in the Ob among the Siberian rivers.

**Figure 5:**
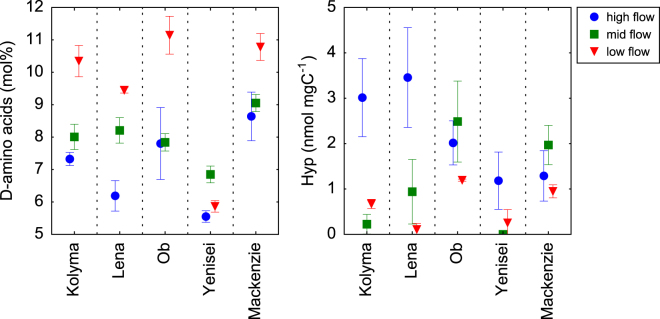
Mol percentages (mol%) of D-amino acids (D-Asx, D-Glx, D-Ser, D-Ala) and C-normalized concentrations of hydroxyproline (Hyp). Mol% D-amino acids were calculated as the sum of the four measured D-amino acids ((D-Asx, D-Glx, D-Ser, D-Ala) divided by the total sum of THAA without glycine and multiplied by 100.

Mol percentages of D-amino acids peaked during low flow conditions across all rivers with exception of the Yenisei (Fig. 5). DOC-normalized concentrations of Hyp, a proxy for the input of plant-derived protein, showed the opposite trend peaking during the Spring freshet, and they were low or below the limit of quantification during Summer/Fall and Winter/early Spring (Fig. 5). Concentrations of Hyp were lowest in the Yenisei and Mackenzie.

Characteristic ratios of D-amino acids provided clues on the macromolecular origin of bacterial DOM^32–34^. Molar ratios of D-Ala:D-Asx and D-Ala:D-Glx and in bacterial DOM from coastal ecosystems and groundwater were 0.7–1.3 and 1.2–2.0, respectively^34,35^ (Table S2).

D-Ala is more widely distributed among biopolymers than other D-amino acids, including D-Ala-rich teichoic acids derived from Gram-positive bacteria^34^. Thus, D-Ala yields exceeding D-Asx and D-Glx by more than two-fold likely indicate increasing contributions of specific bacterial sources in particular teichoic acids.

**Figure 6:**
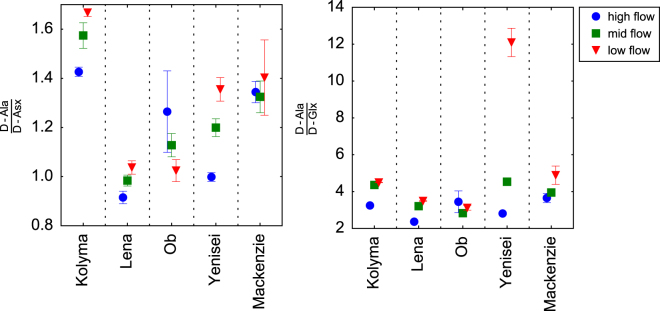
Ratios of D-alanine (D-Ala) to D-aspartic acid (D-Asx) and D-glutamic acid (D-Glx).

D-Ala:D-Asx ratios were lowest (0.9–1.4) during the Spring Freshet, and highest during the Winter/early Spring period (1.0–1.7, Fig. 6). D-Ala:D-Glx ratios followed a similar trend, ranging from 2.4–3.7 during the Spring freshet and 3.1–4.9 during the Winter/early Spring period, with the exception of the Yenisei during Winter/early Spring where a ratio of 12.1 was observed. Both, D-Ala:D-Asx and D-Ala:D-Glx ratios suggested relatively similar macromolecular sources of bacterial DOM as observed in coastal ecosystems and groundwater, and they indicated limited contributions of D-Ala rich macromolecules such as teichoic acids.

**Figure 7:**
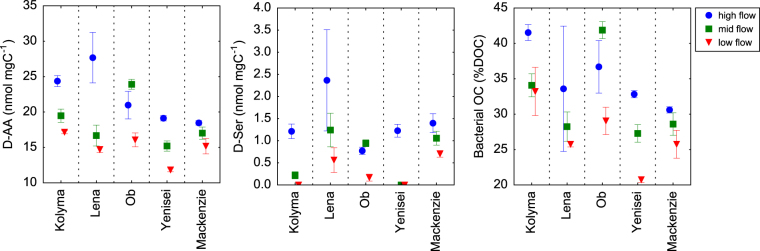
C-normalized concentrations of D-amino acid, D-serine (D-Ser), and percentages of dissolved organic carbon (DOC) derived from bacteria. Bacterial contributions to DOC were calculated on the basis of C-normalized yields of D-glutamic acid (D-Glx), D-alanine (D-Ala), and D-aspartic acid (D-Asx)^34^.

DOC-normalized concentrations of D-amino acids varied seasonally in all rivers with highest concentrations during the Spring freshet. Among D-amino acids, D-Ser exhibited strongest seasonal changes occurring almost exclusively through the Spring freshet (Fig. 7). The percent of DOC derived from bacteria calculated on the basis of D-Ala, D-Asx, and D-Glx abundance showed highest contributions during the Spring freshet and declined during the remainder of the year (Fig. 7). During the Spring freshet 31–42% of DOC was derived from bacteria, whereas during Winter/early Spring 21–33% of DOC was from bacteria (Fig. 7).

## Discussion

Previous studies found the reactivity of DOM in Alaskan Rivers and the Kolyma was strongly linked to the seasonal flow regime^11,24^. Our biochemical data confirmed that all Siberian rivers carried pulses of bioavailable DOM during the Spring freshet and DOM of lower bioavailability for the remainder of the year. Seasonal variability in DOM quality was closely linked to its chemical composition. DOC-normalized yields of major biochemicals including carbohydrates, amino acids and plant phenols across all watersheds were 3–4 times higher during the Spring freshet compared to low flow conditions in Winter. These three classes of biochemicals represent the majority of terrestrial biomass and are reactive substrates supporting aquatic food webs and contributing significantly to carbon dioxide fluxes in rivers^36,37^. Additional compositional features of freshet DOM included low mol % D-amino acids and low mol % glycine, both sensitive indicators of bioavailable DOM^38,39^.

Biochemical trends in the Mackenzie diverged from the patterns observed in the Siberian rivers with no distinct pulses of bioavailable DOC during the freshet periods. This suggested watershed characteristics exerted a strong control on the amount of DOC exported throughout the year. Extensive leaching of surface soils in combination with limited microbial activity during Winter, and high hydrologic connectivity during the freshet explains DOC export in the Siberian rivers^10,40^. Oxygen and hydrogen isotope measurements showed water residence time in the Mackenzie was longest owing to the abundance of large lakes and other water bodies and allowing microbes to remove a large portion of bioavailable DOM^41^.

Yields of biomolecules varied across rivers during the freshet indicating the influence of physical and chemical watershed features on DOM compositions during the Spring flood. Differences were most pronounced in the Kolyma and Mackenzie, both rivers with unique watershed characteristics^10,42^. Dissolved organic carbon in the Kolyma contained higher contributions of carbohydrates than DOC in all other rivers. The Kolyma basin is almost entirely underlain by permafrost and contains carbon-rich Pleistocene loess deposits^42,43^. Extreme bioavailability of permafrost DOC in the Kolyma watershed featuring carbohydrate-like and aliphatic molecular identities was observed, and the high yield of carbohydrates could be indicative of such DOM^44,45^. Kolyma DOC was modern (Δ^14^C-DOC = 47 ± 17) throughout the year, but the presence of a small pool of old DOC could fit within the constraints of endmember values for modern and permafrost DOC (~18,000 years)^12^. Another possibility is that dominated larch forests and shrubland vegetation lead to the higher content of carbohydrates in the DOM.

Dissolved organic carbon in the Ob showed lowest contributions of TDLP among the Siberian rivers consistent with the distribution of major vegetation types within the watershed. The Ob drains one of the largest peat bog system in the world that is dominated by sphagnum mosses with only occasional vascular plant vegetation within the lower reach of the watershed^46^. Only in the middle and upper reaches of the watershed vascular plant vegetation is abundant with strong representations of birch, spruce, and pine forests.

Bioavailable DOM discharged during the freshet already showed heterotrophic processing that must have occurred within the soil, during leaching of litter or during transport in the upper reaches of the watersheds. Neutral sugar compositions in dominant plants types in polar ecosystems substantially differ allowing distinction of vascular plant, moss and lichen sources^47,48^. The Ob watershed contains expansive peat bogs whereas other river watersheds show more diverse vegetation ranging from boreal forests, grasslands or moss-rich wetlands^10^. Relatively uniform neutral sugar compositions across all rivers clearly indicated early stages of degradation. In addition, the bacterial imprint on DOM compositions at all stages of the flow regime was reflected by high abundances of D-amino acids.

Bacterial community structure determined in the same samples showed distinct seasonal shifts that correlated with hydrology and water chemistry in all rivers^49^. Characteristic microorganisms observed during the Spring freshet included Gram-negative bacteria with strong representations from Betaproteobacteria and Bacteriodetes, and smaller contributions from Gram-positive bacteria specifically Actinobacteria^49^. The observed shifts in bacterial assemblages correlated well with characteristic seasonal distributions of D-amino acids. High abundances of D-Ala, D-Asx, D-Glx throughout the year demonstrated the presence of heterotrophic bacteria and bacterially-derived detritus. The regular appearance of D-Ser during the Spring freshet indicated a distinct change of the bacterial community that reassembled each year.

Bacterial communities respond to physico-chemical conditions, and so bacterial activity and diversity is closely linked to the biogeochemistry of aquatic ecosystems^50^. Both, bacterial activity and diversity strongly react to the supply of bioreactive DOM^51,52^, an event occurring annually in the large Arctic rivers during the Spring freshet. The observation of bacterial communities following shifts in flow regime and compositions of organic matter has important consequences for river biogeochemistry and the cycling of organic matter in Artic watersheds. Heterotrophic bacteria rapidly mineralize plant and soil-derived organic matter to CO_2_ and also transform these substrates to new forms of organic matter with varying degrees of bioavailability.

The contribution of bacteria to DOC ranged from 21–42% across all watersheds. For comparison, lignin-based projections showed 16–87% of the DOC in these rivers was derived from vascular plants^10^ suggesting DOM sources were a mixture of plant, soil and bacterially-derived sources. Bacterial DOC estimates were comparable to marine environments (16–30%)^34^ and groundwater (15–34%)^35^. Multiple biomarkers occurring in various bacterial macromolecules were employed to narrow potential errors associated with variable bacterial communities and sources. Endmembers for bacterial DOC were derived from experiments performed in riverine-influenced coastal ecosystems and groundwater^33–35^.

The macromolecular origin of bacterially-derived DOM in rivers appeared to be fairly similar to bacterially-derived DOM in previous experiments^33–35^ although input of potentially Gram-positive soil-derived bacterial components such as teichoic acids especially during the low flow regime was detected. Amon *et al*.^10^ observed a more dominant DOM contribution from deeper soil layers during the low flow seasons. Inputs of DOM from Gram-positive bacteria could overestimate bacterial contributions to DOM affecting most strongly values calculated for the low flow regime. Instead, estimates of bacterially-derived DOM were always lower during low flow than high flow periods across all rivers. This suggested the applied approach lead to relatively robust estimates of bacterial contributions.

Higher bacterial contributions to freshet DOM potentially indicated the production of fresh bacterially-derived DOM. Carbon-normalized D-amino acid concentrations peaked on the downslope of high discharge synchronous with higher concentrations of Hyp in the Ob and Mackenzie. Bacterial metabolism during this period should have been stimulated considering a rich supply of bioavailable DOM and a reactive plant-derived nitrogen source. The production of bacterially-derived DOM with varying degrees of bioavailability is consistent with results from incubation studies and processes observed in the mesopelagic zone of the ocean. Kawasaki and Benner (2006)^33^ observed release of bacterially-derived DOM during growth that was quickly consumed. Kaiser and Benner^34^ found ~65% of bacterially-derived DOM produced in the surface ocean was bioavailable and had a higher reactivity than bulk DOC. Bacterial metabolism is also connected to the production of refractory organic carbon, an observation that has been summarized in the “microbial carbon pump” concept^34,53,54^.

The large bacterial DOC source predicts that bacteria are even more important sources of DON, as they are rich in N-bearing molecules such as amino acids and nucleic acids. For example, amino acids comprised 22–38% of DON in the studied watersheds. We did not have reliable N-normalized yields of bacterial biomarkers to calculate bacterial contributions to riverine DON. In the marine environment, Kaiser and Benner^34^ calculated 47–54% of DON was derived from bacteria, and similar contributions are expected to occur in Arctic watersheds. In addition to bacterially-derived DON, Hyp indicated plant proteins were important sources of DON during the freshet. Vegetation type was identified as a driver of DON exported from Arctic watersheds^55^. C-normalized concentrations of Hyp were fairly similar across the Siberian rivers suggesting the release of plant-derived proteins was controlled by bacterial transformations and uptake.

In essence, bacterial sources were important sources of DON throughout the year through leaching of soils, microbial leaching of plant detritus, and transformations of DOM within the river channels^16^. Additionally, plant-derived proteins were seasonally important sources of DON specifically during the Spring freshet. Bioavailable DON from both sources should help meet N demand in coastal seas after discharge. Although the overall N subsidy from rivers to the Arctic Ocean is considered to be limited, the potential for supporting estuarine and near-shore productivity is well recognized^15^.

The observed molecular and chemical features present a unifying concept for DOM cycling in Arctic watersheds and a better understanding of how bacterial metabolism is linked to DOM composition and carbon fluxes. The export of bioavailable DOC and DON from Arctic watersheds is connected to river morphology, dominant vegetation and soil characteristics. Similar to the marine ecosystems, bacterial metabolism and transformations shape bioactive element stoichiometries, the chemical compositions of organic matter, and export of carbon in Arctic watersheds.

The important contribution of bacterial detritus to riverine DOC alludes to high bacterial activity in these watersheds year round. Extensive remineralization and transformations of DOC already occur in the upper reaches of the watersheds, for example erasing the old radiocarbon permafrost signature in exported DOC in the Kolyma^45^. Applying terrigenous DOC decay constants of Kaiser *et al*.^26^ a transport time of a few weeks from drainage networks to the mouth of the river where samples were collected would result in up to 40% loss of riverine DOC. This indicates a large portion of freshly mobilized DOC is rapidly lost in the upper reaches of the watersheds associated with substantial CO_2_ release that is not accounted for in current carbon budgets^56^. As the Arctic regions are projected to experience substantial warming affecting permafrost stability, increase freshwater runoff, shift in dominant vegetation and increased primary productivity, it is essential to study upper stream networks to more accurately capture the effect of a changing climate on biogeochemical cycles in Arctic watersheds.

## Methods

Sample from five major Arctic rivers were sampled by the Pan-Arctic River Transport of Nutrients, Organic Matter, and Suspended Sediments (PARTNERS) project between 2003–2007. Samples were collected close to the river mouth for each river (Fig. S4). Raymond *et al*.^3^, McClelland *et al*.^57^, and Holmes *et al*.^4^ described detailed sampling programs and procedures. The PARTNERS collection protocol was designed to capture base flow (under ice), Spring melt, and late summer conditions. The timing of Spring sampling was determined by physical access to sampling sites immediately after the ice break-up. The collection device was a torpedo shaped, Teflon coated, 60 kg depth-integrated sampler (US D-96). The rivers were sampled at five different locations along a cross-channel transect and combined into one homogeneous sample using a Teflon churn. With the exception of winter samples, which were collected by drilling a hole in the ice, each water sample is representative not only of surface to bottom, but cross-channel chemistry. Water from the Teflon churn was then filtered (0.45 µm Pall Aquaprep 600 capsule filters) into acid washed 1 L polycarbonate bottles and frozen.

DOC and total dissolved nitrogen (TDN) were measured by high temperature combustion using a Shimadzu TOC-V analyzer.

### Total and D/L-amino acids

Total amino acids including hydroxyproline (Hyp), histidine (His), serine (Ser), arginine (Arg), glycine (Gly), aspartic acid (Asx), glutamic acid (Glx), threonine (Thr), alanine (Ala), lysine (Lys), tyrosine (Tyr), methionine (Met), valine (Val), isoleucine (Ile), leucine (Leu), phenylalanine (Phe) were analyzed after vapor phase hydrolysis by liquid chromatography and fluorometric detection. The hydrolysis was performed according to Kaiser and Benner^58^. Primary amino acids were derivatized with o-phthaldialdehyde (OPA) and mercaptopropionic acid (MPA), and secondary amino acids were derivatized with 9-fluorenylmethyl chloroformate (FMOC) for separation and detection. The OPA reagent was made from 630 µL OPA stock (36 mg OPA in 2.5 mL methanol) mixed with 22 µL of MPA dissolved into 0.5 M sodium borate buffer (pH = 10.2). The FMOC reagent was prepared by dissolving 42 mg of FMOC into 1.0 mL of acetonitrile. After derivatization and before injection the pH was adjusted to 7 by adding diluent (pH = 1.5) that was prepared with 33 mL of mobile phase A and 0.5 mL of concentrated phosphoric acid. The column was a Zorbax Eclipse Plus C18 Rapid Resolution HT column (4.6 × 50mm 1.8 µm) with guard. Mobile phase A was 9.7 mmol L^−1^ K_2_HPO_4_ and 9.7 mmol L^−1^ boric acid adjusted to 8.15 with 50 w/w % NaOH. Mobile phase B was acetonitrile: methanol (MeOH): MQ water (45:45:10 v/v). Separation of amino acids was achieved at 40 °C with a linear gradient starting with 98% A isocratically for 2 min., then 43% A after 7 minutes. The column was flushed with 100%B for 0.5 min after 7 min. and then returned to starting conditions. For detection the detector was set to excitation at 230 nm and emission at 450 for 5.7 min then switch to excitation at 266 nm and emission at 305 nm. The flow rate was 2 mL min^−1^, and total run time was 9.4 minutes.

D- and L-amino acids were analyzed according to Kaiser and Benner^58^. Briefly, after hydrolysis and neutralization, amino-acid enantiomers were derivatized with a mixture of N-isobutyryl-L-cysteine and o-phthaldialdehyde or N-isobutyryl-D-cysteine and o-phthaldialdehyde and separated on a reversed-phase column. Samples were run with both reagents to allow for correction of co-eluting peaks. Measured values of enantiomeric amino acids were corrected for acid-catalyzed racemization using the mean of the racemization observed in proteins and free amino acids^58^. Aspartic acid and glutamic acid were reported as Asx and Glx because acid hydrolysis converted asparagine and glutamine to the respective amino acid and so concentrations reflected both amino acids.

### Neutral Sugar Analysis

Seven neutral sugars (fucose (Fuc), rhamnose (Rha), arabinose (Ara), galactose (Gal), glucose (Glc), mannose (Man), xylose (Xyl)) are analyzed according to Skoog and Benner^59^ with modifications. Briefly, samples were hydrolyzed in 1.2 mol L^−1^ sulfuric acid and neutralized with a self-absorbed ion retardation resin^60^. After desalting with a mixture of cation and anion exchange resins, neutral sugars were isocratically separated with 25 mM NaOH on a PA 1 column in a Dionex 500 system with a PAD. Detector settings were analogous to Skoog and Benner^59^.

### Annual Load Calculations

Annual loads for THAA and THNS for each river were calculated using the US Geological Survey LoadEstimator (LOADEST) program coupled with the LoadRunner interface to automate runs^61,62^. Daily discharge data between 2003 and 2007 for the five Arctic rivers in this study were obtained from the Arctic Great Observatory and PARTNERS websites. Annual load outputs were converted to Tg C yr^−1^.

### Bacterial contribution to riverine DOM

Bacterial processing of riverine DOM is reflected by C-normalized concentrations of D-Asx, D-Glx, D-Ser, and D-Ala^21,34^. Contributions of bacteria to carbon in DOM were determined by comparing C-normalized biomarker yields in field samples to average yields in freshly-produced bacterial DOM^33,34^. The percentages of bacterial C in riverine DOM were calculated as follows:

$$Bacterial\,DOC\,( \% )=\frac{biomarke{r}_{DOC}}{biomarke{r}_{bacterialDOC}}\ast 100$$

where Biomarker_DOM_ is the C- normalized concentration of a specific biomarker in riverine DOM and Biomarker_bacterial DOM_ is the C-normalized concentration in bacterial DOM. Average D-amino acid yields of D-Asx, D-Glx, and D-Ala (nmol mgC^−1^) from incubation experiments in two freshwater systems and groundwater were used as endmembers for 100% bacterial DOM (Table S4). D-Ser was excluded in the calculation because it’s distribution was very variable in bacterial cells and bacterial DOM and usually showed distinct bacterial populations^34^. Biomarker yields for bacterial DOC used for calculations were 16.2 ± 9.0 D-Asx, 15.5 ± 1.9 D-Glx, and 22.2 ± 5.9 D-Ala.

## Electronic supplementary material

Supplemental information
