# Supplementary material for: Origins and transformations of dissolved organic matter in large Arctic rivers
Source: Sci Rep. 2017 Oct 19;7:13064. doi: 10.1038/s41598-017-12729-1 (PMC5648890; doi:10.1038/s41598-017-12729-1)
Supplement: Supplementary file 1 — Supplemental information [file 41598_2017_12729_MOESM1_ESM.pdf]

[Nature – Scientific Reports]

Supporting Information for

[Origins and transformations of dissolved organic matter in large Arctic rivers]

[Karl Kaiser[1,2]*,Maria Canedo-Oropeza[2],Rachel McMahon[3], Rainer Amon[1,2]]

[[1]Texas A&M University Galveston Campus, Galveston, TX 77444, Department of Marine Sciences, [2]Texas A&M University, College Station, Department of Oceanography, [3]Old Dominion University, Norfolk, Department of Ocean, Earth and Atmospheric Science]

Contents of this file

Tables S1 to S4
Figures S1 to S4

Introduction

Supporting data presented in tables below show detailed biochemical data in river samples, information on dominant vegetation, and D-amino acid biomarker data used for calculation of bacterial DOC content.

Supporting figures show information on sampling location, chemical composition, and correlation with lignin phenols.

Table S1: Concentrations and carbon-normalized yields of total hydrolysable neutral sugars (THNS).

River	Date	Discharge $m^3 s^{-1}$	THNS yield (%OC)	Fuc	Rha	Rha	Gal	Glc	Man	Xyl	THNS sum
				(nmol L^{-1})							
Kolyma	6/11/2004	17300	3.4	422	715	518	803	1153	803	693	5108
Kolyma	6/25/2004	11700	1.9	163	226	191	243	446	242	247	1759
Kolyma	7/15/2004	7140	1.2	110	135	121	142	253	135	169	1065
Kolyma	8/10/2004	4860	1.0	63	74	57	92	121	79	91	577
Kolyma	8/25/2004	5970	0.4	36	45	36	15	61	41	33	267
Kolyma	4/22/2005	200.83	0.5	27	33	31	29	52	29	33	234
Kolyma	7/19/2005	4004.19	1.0	56	68	61	70	156	90	138	639
Kolyma	8/14/2005	4505.78	0.8	104	104	54	143	189	59	57	710
Kolyma	8/27/2005	2901.84	0.6	65	93	81	108	144	105	109	705
Kolyma	7/24/2006	NA	0.5	57	87	56	86	158	61	73	578
Lena	8/12/2003	19100	0.6	61	75	64	68	73	71	69	481
Lena	4/9/2004	2275	0.9	92	137	122	108	209	116	113	896
Lena	6/5/2004	61408	2.9	556	888	590	1005	1357	1114	774	6284
Lena	6/7/2004	90720	1.1	143	513	255	398	259	230	256	2053
Lena	8/19/2004	35500	1.5	122	192	139	211	335	224	186	1408
Lena	8/24/2004	31100	0.9	113	53	115	159	244	86	122	891
Lena	10/7/2004	24800	0.6	69	51	88	81	154	48	81	573
Lena	10/10/2004	26188.8	0.5	56	104	65	79	115	77	74	571
Lena	3/24/2005	3946.9	0.5	63	76	54	86	175	89	81	624
Lena	5/27/2005	53784	2.5	570	524	610	878	1369	682	719	5351
Lena	6/4/2005	109800	1.0	248	221	258	345	581	265	279	2197
Lena	8/14/2005	37700	0.6	77	102	78	108	166	100	97	729
Lena	10/9/2005	23900	0.8	78	72	121	151	220	136	117	895
Lena	6/6/2006	89100	3.9	610	982	750	1200	1881	1327	953	7703
Mackenzie	3/24/2004	3780	0.5	51	40	28	38	13	31	47	248
Mackenzie	6/17/2004	18300	0.7	75	78	57	80	98	74	84	547
Mackenzie	6/22/2004	17400	0.5	54	49	36	60	30	39	53	323
Mackenzie	7/13/2004	12500	0.8	38	61	30	39	192	25	39	425
Mackenzie	8/4/2004	12400	0.3	26	24	20	23	34	15	24	166
Mackenzie	8/25/2004	8730	0.8	61	30	37	70	90	32	70	389
Mackenzie	3/16/2005	3670	0.4	47	34	26	37	16	32	46	238
Mackenzie	6/14/2005	25200	0.3	29	35	26	6	54	32	24	206
Mackenzie	6/29/2005	16900	0.6	64	66	43	64	59	54	69	420
Mackenzie	8/9/2005	13500	0.4	43	40	30	41	50	26	39	269
Mackenzie	8/30/2005	12300	0.4	68	36	34	65	50	35	65	353
Mackenzie	8/15/2006	12700	0.3	36	31	24	4	75	26	24	220
Mackenzie	3/27/2007	2960	0.3	58	31	24	50	14	28	49	253
Ob	4/5/2004	3692.46	0.8	66	79	43	66	217	66	68	605
Ob	6/15/2004	34500	1.8	239	335	212	356	480	314	331	2267
Ob	6/17/2004	34500	1.8	222	275	174	364	568	345	296	2245
Ob	7/28/2004	32700	0.5	101	120	89	129	137	90	123	789
Ob	8/11/2004	15900	1.4	171	316	196	309	487	324	253	2056
Ob	10/11/2004	8920	1.0	148	166	84	198	372	129	155	1251
Ob	10/14/2004	9600	0.7	102	131	59	111	236	95	105	839
Ob	3/15/2005	4048.61	0.5	71	101	48	68	149	56	68	562
Ob	6/4/2005	34800	1.9	260	379	284	409	577	354	394	2657
Ob	6/6/2005	34800	1.7	256	364	259	395	505	339	389	2506
Ob	6/28/2005	29200	1.3	216	271	222	312	388	241	321	1971
Ob	7/14/2005	26600	1.3	232	268	228	313	425	231	308	2004
Ob	9/5/2005	9760	1.7	222	224	101	327	1215	203	211	2504
Ob	6/7/2006	32949.23	2.1	271	393	259	477	760	464	349	2972

Ob	11/23/2006	5814	1.1	119	181	94	148	802	131	170	1645
Yenisei	3/19/2004	7360	1.4	67	92	49	83	143	71	86	592
Yenisei	6/14/2004	98500	1.4	198	335	232	426	645	452	337	2625
Yenisei	8/25/2004	18200	1.0	92	90	53	114	148	83	117	697
Yenisei	10/1/2004	19880	0.3	37	45	35	52	59	41	46	315
Yenisei	6/11/2005	78800	2.5	269	428	353	524	840	542	454	3410
Yenisei	6/16/2005	54900	2.0	333	399	288	477	520	432	411	2861
Yenisei	6/17/2005	51500	2.2	342	281	304	488	703	325	472	2915
Yenisei	8/21/2005	14150	0.4	55	53	35	36	74	48	46	346
Yenisei	9/21/2005	15310	0.8	87	92	61	106	125	81	86	638
Yenisei	6/17/2006	82200	1.7	270	415	301	523	866	440	442	3257
Yenisei	11/22/2006	23245.55	0.4	30	34	22	33	34	34	40	226

Table S2. Concentrations, carbon-normalized yields and mole percentages of D-amino acids (mol%) of total hydrolysable amino acids (THAA).

River	Date	Discharge (m^3 s^{-1})	THAA yield (% OC)	Asp	Glu	Ser	His	Thr	Gly	Arg	Ala	Tyr	Val	Ile	Leu	Lys	Hyp	THAA	%D
				(nmol L^{-1})															
Kolyma	8/26/2003	1820	1.2	111	78	73	23	72	297	8	125	2	34	1	23	13	2	863	10.7
Kolyma	6/11/2004	17300	2.5	840	566	537	53	625	1575	59	829	43	377	121	254	91	46	6017	7.0
Kolyma	6/15/2004	15020	2.3	666	449	416	45	475	1310	53	648	37	284	89	190	70	15	4746	7.5
Kolyma	6/25/2004	11700	2.0	398	288	242	37	273	832	37	417	13	171	42	119	48	13	2930	7.9
Kolyma	7/15/2004	7140	1.6	322	229	187	30	195	713	26	331	1	121	26	83	30	0	2293	9.4
Kolyma	8/10/2004	4860	2.0	238	218	167	29	145	527	24	264	6	97	20	75	26	8	1844	7.7
Kolyma	8/25/2004	5970	2.0	280	244	207	30	168	623	26	290	1	108	24	79	27	0	2107	7.7
Kolyma	9/23/2004	3000	1.8	214	175	153	28	131	527	20	237	6	82	16	66	20	0	1674	8.4
Kolyma	4/22/2005	201	1.6	130	98	74	14	78	295	13	142	4	50	140	39	14	0	1091	7.2
Kolyma	7/19/2005	4004	1.5	222	155	130	32	107	503	20	212	0	79	17	60	31	0	1568	7.0
Kolyma	8/14/2005	4506	1.3	273	177	143	32	166	630	18	268	0	96	19	67	31	0	1920	7.5
Kolyma	8/27/2005	2902	1.5	425	279	221	40	255	858	23	387	7	156	38	106	49	0	2844	7.2
Kolyma	7/24/2006	NA	1.8	419	382	341	46	244	1102	50	399	101	153	42	128	61	0	3469	5.9
Kolyma	11/20/2006	NA	0.9	209	142	126	31	110	561	21	192	56	67	13	45	23	0	1596	6.2
Lena	8/12/2003	19100	0.8	125	78	73	22	75	409	15	132	3	35	11	28	44	0	1051	9.0
Lena	4/9/2004	2275	1.2	293	168	159	20	143	641	33	240	2	78	23	59	56	1	1917	10.4
Lena	6/5/2004	61408	2.9	1574	955	909	58	950	2387	153	1257	88	589	203	402	222	63	9810	8.2
Lena	8/19/2004	35500	1.8	427	315	218	24	217	750	62	361	17	129	45	107	97	0	2769	9.1
Lena	8/24/2004	31100	1.7	388	269	206	26	194	656	54	334	21	128	49	115	88	0	2526	7.5
Lena	10/7/2004	24800	1.3	310	200	160	22	141	605	36	249	12	80	28	62	66	40	2011	9.7
Lena	10/10/2004	26189	0.9	250	152	142	22	114	548	30	193	10	63	23	49	55	0	1651	9.8
Lena	3/24/2005	3947	1.1	356	172	185	22	186	738	38	293	12	73	22	51	57	11	2217	10.0
Lena	5/27/2005	53784	2.9	1400	1162	1086	35	872	2355	143	1241	78	537	197	378	173	69	9728	5.1
Lena	6/4/2005	109800	0.8	433	268	246	10	257	762	41	353	21	155	52	99	45	0	2742	5.9
Lena	8/6/2005	45500	1.3	418	295	220	5	209	822	48	365	15	140	42	95	51	5	2729	7.4
Lena	8/14/2005	37700	1.3	372	280	220	5	177	798	53	324	18	117	43	93	54	0	2554	7.0
Lena	10/10/2005	23200	0.9	238	148	103	2	113	537	23	211	5	70	19	44	26	0	1537	8.3
Lena	6/6/2006	89100	2.6	1317	799	749	41	790	2199	101	1064	64	482	175	329	145	61	8316	5.8
Lena	11/14/2006	43400	0.9	301	170	144	4	148	756	27	287	9	89	23	53	29	0	2040	9.4
Mackenzie	3/24/2004	3780	0.9	115	74	70	0	49	334	10	125	0	32	12	18	11	3	853	10.3
Mackenzie	6/17/2004	18300	2.2	309	300	337	15	140	929	49	289	24	92	59	103	53	10	2709	5.4
Mackenzie	6/22/2004	17400	1.1	184	120	94	0	86	407	19	165	7	55	22	39	21	0	1218	10.1
Mackenzie	7/13/2004	12500	1.2	149	113	99	0	70	359	10	151	8	45	17	29	15	11	1076	9.5
Mackenzie	8/4/2004	12400	1.0	129	80	65	0	63	303	9	122	0	37	14	25	10	13	870	9.4
Mackenzie	8/25/2004	8730	1.2	136	102	81	1	66	313	15	144	5	40	16	29	16	7	971	8.6
Mackenzie	3/16/2005	3670	0.7	106	63	61	0	50	308	12	116	0	25	14	17	12	3	786	10.2
Mackenzie	6/14/2005	25200	1.1	197	130	98	50	100	432	12	184	9	60	5	42	18	12	1349	9.2
Mackenzie	6/29/2005	16900	1.2	183	117	99	49	94	439	10	191	9	56	3	40	15	0	1303	9.3

Mackenzie	7/14/2005	16200	1.1	184	124	88	48	94	420	14	191	11	53	4	42	12	0	1286	9.4
Mackenzie	8/9/2005	13500	1.1	169	133	111	0	87	397	11	178	10	52	2	39	12	5	1207	8.9
Mackenzie	8/30/2005	12300	0.9	174	146	125	48	94	409	12	183	0	51	3	39	14	5	1302	8.2
Mackenzie	8/15/2006	12700	0.9	159	100	71	0	82	373	11	169	9	45	0	37	12	9	1077	9.8
Mackenzie	3/27/2007	2960	0.6	114	69	55	0	57	327	6	146	0	28	0	22	4	7	835	11.7
Ob	7/16/2003	31200	1.4	535	350	352	32	323	1329	39	514	19	174	48	92	48	12	3868	11.9
Ob	4/5/2004	3692	1.3	215	187	136	18	130	527	22	263	5	82	21	52	26	6	1688	10.1
Ob	6/17/2004	34500	2.3	652	525	415	35	405	1279	78	636	29	258	83	171	88	29	4683	8.5
Ob	7/28/2004	32700	1.9	726	581	494	31	397	1569	95	725	49	276	96	206	92	59	5395	8.6
Ob	8/11/2004	15900	2.5	761	690	462	35	447	1409	110	812	64	331	121	272	122	9	5644	7.9
Ob	10/11/2004	8920	1.7	464	400	261	24	258	867	68	457	23	180	60	140	61	29	3293	7.9
Ob	10/14/2004	9600	1.8	503	426	287	24	267	990	75	491	25	181	60	134	61	0	3525	8.7
Ob	3/15/2005	4049	1.0	261	208	133	19	141	671	18	274	8	77	19	41	19	9	1897	12.9
Ob	6/4/2005	34800	2.2	681	566	419	22	448	1342	86	662	25	286	97	217	88	24	4962	4.6
Ob	6/6/2005	34800	2.0	670	507	386	22	448	1346	82	653	27	280	104	198	76	0	4800	7.6
Ob	6/28/2005	29200	2.1	676	542	419	25	430	1416	79	697	31	269	93	195	86	40	4998	7.7
Ob	7/14/2005	26600	2.3	787	659	470	27	484	1590	98	805	53	332	125	251	104	0	5786	7.8
Ob	9/5/2005	9760	2.7	790	736	496	25	478	1522	137	845	61	364	139	327	126	7	6054	6.1
Ob	9/17/2005	8890	2.6	793	723	461	23	438	1410	168	833	67	351	133	306	128	16	5851	6.0
Ob	6/7/2006	32949	2.0	616	508	379	29	406	1203	83	620	9	271	90	208	87	26	4533	6.4
Ob	11/23/2006	5814	1.1	388	273	213	16	201	997	49	355	1	123	32	94	37	13	2792	10.6
Yenisei	3/19/2004	7360	1.4	113	125	115	0	59	359	11	141	3	42	14	29	13	4	1028	5.2
Yenisei	6/14/2004	98500	2.3	1015	714	657	58	603	1820	74	843	46	395	140	264	89	32	6751	5.8
Yenisei	6/16/2004	94500	2.7	1057	857	915	106	643	1964	63	927	49	426	144	281	131	45	7607	4.8
Yenisei	6/18/2004	90600	2.0	832	520	473	48	477	1493	57	674	36	319	105	206	81	0	5322	6.1
Yenisei	8/25/2004	18200	1.8	276	237	210	30	163	609	35	295	4	104	33	78	41	0	2113	5.8
Yenisei	10/1/2004	19880	1.2	289	187	143	30	128	651	27	264	1	93	23	63	34	0	1934	7.3
Yenisei	10/2/2004	19370	1.2	299	187	141	32	148	659	23	272	4	100	24	64	35	0	1988	7.6
Yenisei	6/11/2005	78800	2.3	762	493	457	44	455	1348	61	639	28	306	108	210	91	0	5004	5.5
Yenisei	6/16/2005	54900	2.0	668	439	401	45	390	1232	62	570	33	267	93	186	87	0	4473	5.8
Yenisei	6/17/2005	51500	2.0	640	420	389	46	383	1194	53	566	28	254	91	176	81	0	4321	5.6
Yenisei	8/16/2005	14290	1.6	286	228	165	32	160	625	31	285	0	105	32	82	45	0	2076	6.7
Yenisei	8/21/2005	14150	1.5	287	229	167	33	167	615	39	292	6	111	36	84	49	0	2115	6.2
Yenisei	9/21/2005	15310	1.5	264	188	153	32	145	589	24	249	5	87	30	67	36	0	1867	6.7
Yenisei	6/17/2006	82200	2.2	986	623	608	59	601	1754	81	810	48	401	136	266	117	0	6491	5.5
Yenisei	11/22/2006	23246	1.2	155	123	102	30	78	389	9	152	0	46	14	34	20	0	1153	6.0

Table S3. Geographical, climatic and geochemical characteristics of river/watershed systems investigated in this project (from Amon et al. 2012).

River/Watershed characteristics	Mackenzie	Ob	Yenisey	Lena	Kolyma
Discharge (km^3 yr^{-1})[1]	298	427	636	581	111
Length (km)[1]	3679	3977	4803	4387	2091
Catchment (10^6 km^2)[1]	1.78	2.99	2.54	2.46	0.65
MAAT (°C)	0.7	1.4	-1.0	-6.5	-10.1
Mean slope (m km^{-1})	2.23	1.28	1.94	1.83	2.16
Sediment flux (10^6 t/y)[2]	124	15.5	4.7	20.7	10.1
Southernmost Lat. (°N)	52.2	45.3	45.7	52.2	60.6
Cont. permafrost (%)[3]	13	1	31	77	99
Deciduous BL forest (%)[3]	1.4	10.2	3.4	1.1	0.4
Evergreen NL forest (%)[3]	23.7	14.9	20.6	7.4	0.2
Deciduous NL forest (%)[3]	0	1.5	32.7	58.8	49.1
Mixed forest (%)[3]	9.2	12.0	10.6	4.9	0.2
Total forest (%)[3]	34.4	38.6	67.3	72.1	49.9
Forest – MODIS (%)[3]	35	25	35	32	10
Shrubland (%)[3]	10.5	2.6	9.0	12.5	32.1
Grassland (%)[3]	30.0	15.9	7.2	0.8	0.1
Cropland (%)[3]	2.4	22.9	6.2	0.6	0
Wetlands (%)[3]	0.1	8.5	2.6	3.3	3.8
Water bodies (%)[3]	10.3	2.4	2.1	1.7	1.6

[1]Holmes et al. 2011, [2]Holmes et al. 2002, [3]From both Modis vegetation continuous fields (VCF) data and Global Land Cover (GLC) data. For reference see: Modis VCF - http://glcf.umiacs.umd.edu/data/vcf/ and GLC - http://ies.jrc.ec.europa.eu/global-land-cover-2000 and http://bioval.jrc.ec.europa.eu/products/glc2000/products.php;
MAAT = mean annual air temperature, BL = broad leaf, NL = needle leaf.

Table S4: Yields of D-amino acids in freshly-produced bacterial dissolved organic matter (Bacterial DOM).

Bacterial DOM	D-Asx	D-Glx	D-Ala	D-Ser	D-Ala/D-Glx	D-Ala/D-Asx
	(nmol mg C^{-1})					
Groundwater[1]	5.8	15.0	24.0	6.6	1.6	4.1
Coasta Atlantic 1[2]	21.8	13.8	15.7	10.5	1.1	0.7
Coastal Atlantic 2[2]	21.1	17.6	27.0	1.6	1.5	1.3

[1] Data from Shen and Benner (2014)
[2] Experiments is described in Kawasaki and Benner (2006) and Kaiser and Benner (2008).

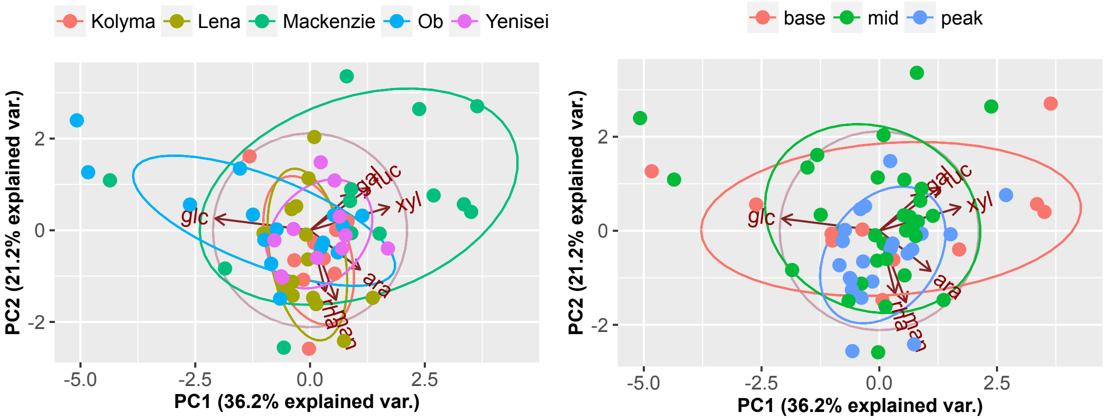

Fig. S1. Principal component analysis showing compositions of neutral sugars in rivers and during different flow regimes. High flow reflects the Spring flood, mid flow the transition period after peak discharge, and low flow represents late Fall, Winter, and early Spring discharge periods. Ellipses describe confidence regions at the 95% level.

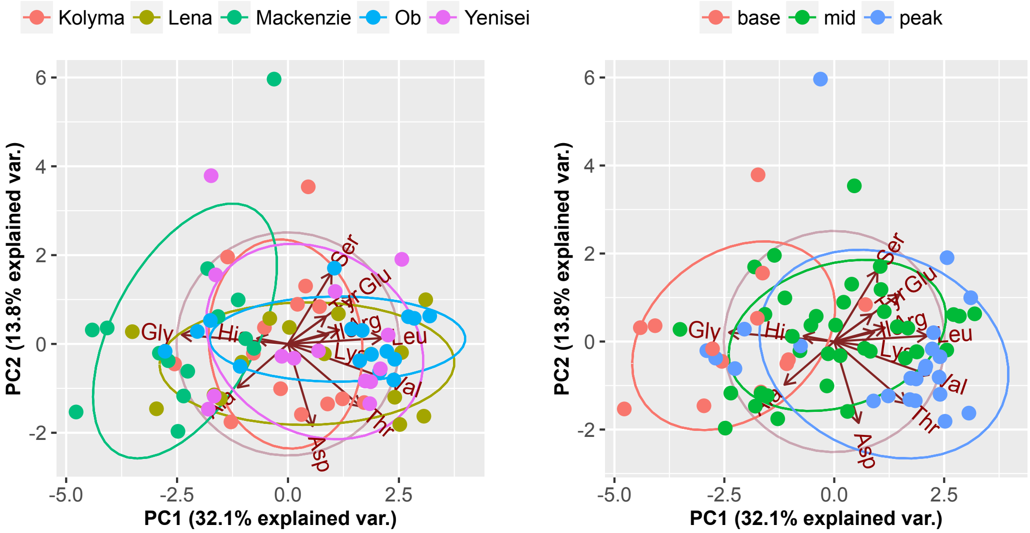

Fig. S2. Principal component analysis showing compositions of amino acids in rivers and during different flow regimes. High flow reflects the Spring flood, mid flow the transition period after peak discharge, and low flow represents late Fall, Winter, and early Spring discharge periods. Ellipses describe confidence regions at the 95% level.

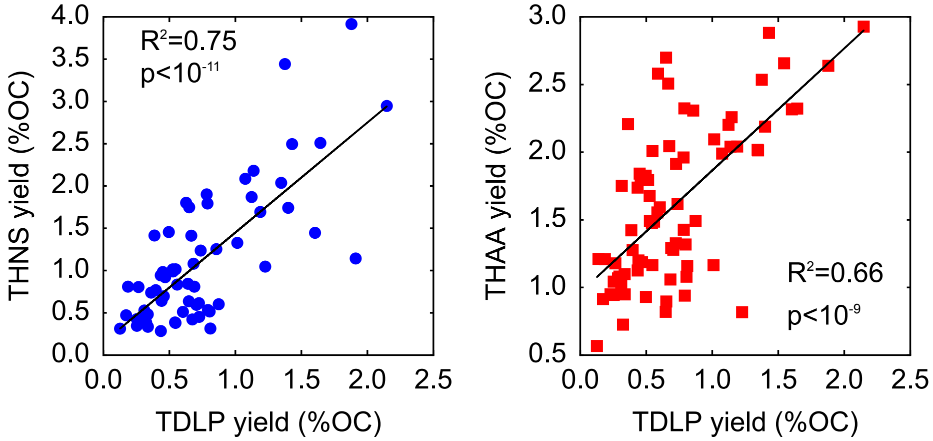

Fig S3. Correlation between total dissolved phenols (TDP) with total hydrolysable neutral sugars (THNS) and total hydrolysable amino acids (THAA). TDP is from Amon et al. (2012).

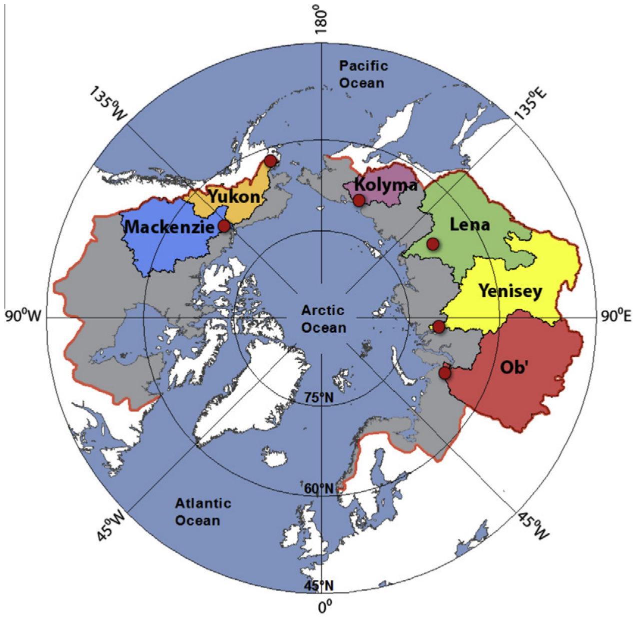

Fig S4: Sampling locations and extend of watersheds of major Arctic rivers. Figure is from Amon et al. (2012).
